# The Immunomodulation of Acetylcholinesterase in Zhikong Scallop *Chlamys farreri*

**DOI:** 10.1371/journal.pone.0030828

**Published:** 2012-01-23

**Authors:** Xiaowei Shi, Zhi Zhou, Lingling Wang, Feng Yue, Mengqiang Wang, Chuanyan Yang, Linsheng Song

**Affiliations:** 1 Key Laboratory of Experimental Marine Biology, Institute of Oceanology, Chinese Academy of Sciences, Qingdao, China; 2 Graduate University of Chinese Academy of Sciences, Beijing, China; Carl-Gustav Carus Technical University-Dresden, Germany

## Abstract

**Background:**

Acetycholinesterase (AChE; EC 3.1.1.7) is an essential hydrolytic enzyme in the cholinergic nervous system, which plays an important role during immunomodulation in vertebrates. Though AChEs have been identified in most invertebrates, the knowledge about immunomodulation function of AChE is still quite meagre in invertebrates.

**Methodology:**

A scallop AChE gene was identified from *Chlamys farreri* (designed as CfAChE), and its open reading frame encoded a polypeptide of 522 amino acids. A signal peptide, an active site triad, the choline binding site and the peripheral anionic sites (PAS) were identified in CfAChE. The recombinant mature polypeptide of CfAChE (rCfAChE) was expressed in *Pichia pastoris* GS115, and its activity was 71.3±1.3 U mg^−1^ to catalyze the hydrolysis of acetylthiocholine iodide. The mRNA transcripts of CfAChE were detected in haemocytes, hepatopancreas, adductor muscle, mantle, gill, kidney and gonad, with the highest expression level in hepatopancreas. The relative expression level of CfAChE mRNA in haemocytes was both up-regulated after LPS (0.5 mg mL^−1^) and human TNF-α (50 ng mL^−1^) stimulations, and it reached the highest level at 12 h (10.4-fold, P<0.05) and 1 h (3.2-fold, P<0.05), respectively. After Dichlorvos (DDVP) (50 mg L^−1^) stimulation, the CfAChE activity in the supernatant of haemolymph decreased significantly from 0.16 U mg^−1^ at 0 h to 0.03 U mg^−1^ at 3 h, while the expression level of lysozyme in the haemocytes was up-regulated and reached the highest level at 6 h, which was 3.0-fold (P<0.05) of that in the blank group.

**Conclusions:**

The results collectively indicated that CfAChE had the acetylcholine-hydrolyzing activity, which was in line with the potential roles of AChE in the neuroimmune system of vertebrates which may help to re-balance the immune system after immune response.

## Introduction

Acetycholinesterase (AChE; EC 3.1.1.7) is an essential hydrolytic enzyme in the cholinergic nervous system, and responsible for catalyzing the degradation of acetylcholine (ACh) into acetate and choline [1]. Because AChE is capable of altering the acetylcholine level in the synaptic cleft and blood plasma, it plays a crucial role in the physiological activity adjustment of the cholinergic nervous system [2]–[5].

AChEs have been so far identified in different tissues of most vertebrates and more than 20 invertebrate animals [6]–[8]. For instance, AChE activity has been detected in erythroid cells [9], brain [10], muscle [10], liver [11], kidney [12] and lungs [13] of vertebrates, And it was also detectable in different tissues of invertebrates [14]–[18], such as in the gills, mantle and haemolymph of mollusc [14], [15], [19], the eye and brain of arthropod [19], and the head of nematode [17], [18]. There is a great difference in the amino acid sequence of AChEs from different animal, and it even varies greatly among the different tissues of the same organism [17]. All the AChEs share some conserved structural features responsible for their catalysis function. For example, an active site triad (Ser, Glu and His) exist in all the reported AChEs, and the three residues form a plannar array at the bottom of a deep and narrow gorge, which closely resembles the catalytic triad of other α/β hydrolase fold family proteins [1], [20]. All the AChEs have peripheral anionic sites (PAS) composed of several conserved residues (Trp, Asp and Tyr) at the rim of the active site triad [21], and PAS could affect the activity of AChEs through altering the catalytic triad conformation [21]–[23]. Furthermore, a conserved Trp residue has been identified as part of the choline binding site near the catalytic triad [2]. Except for conserved amino acid residues, there are usually two or three conserved intramolecular disulfide bonds in AChEs to keep these crucial sites in a correct spatial structure [2]. These conserved sites and disulfides bonds provide not only the essential structural elements for the catalytic function of AChE but also conformational positions for some AChE inhibitors [24]. For instance, organophosphorus pesticide has been demonstrated to bind covalently Ser residue at the active site triad to inhibit irreversibly the catalytic activity of AChE [23]. And when the PAS site is bound by non-competitive inhibitors of AChE such as the peptidic toxin, fasciculin, and propidium, it will block the entry of ACh and the exit of choline from the active site by allosteric alteration of the catalytic triad conformation [1], [25]–[29].

AChE in vertebrates hydrolyzes ACh to regulate the physiological activity of the cholinergic nervous system including the immunomodulation mediated by the nicotinic acetylcholine receptor and cholinergic anti-inflammatory pathway. In the pathway, ACh is able to inhibit the excessive product of proinflammatory cytokines through nicotinic acetylcholine receptors to reduce the immune damage and restore the homeostasis [30]. Therefore, AChE has been considered as an intrinsic regulator of inflammation [30]. Recent researches also have demonstrated that immune challenge, such as LPS stimulation, is able to increase AChE activity significantly in salt soluble fractions of frontal cortex and hypothalamus of mice [31], and striatum, cerebral cortex and hippocampus of rat brain [32]. And the AChE function could also be regulated by a variety of other factors, such as cytokines and AChE inhibitors [33]. In invertebrate, the cholinergic pathway is well characterized in *Drosophila melanogaster* and *Caenorhabditis elegans*, and AChE is found to have effect on egg laying and embryonic development through the modulation of ACh level [31], [34]–[36]. However, there is still no report on the immunomodulation of AChEs in invertebrates.

The scallop *Chlamys farreri* is one of important mollusc species cultured widely in the northern coastal provinces of China. In recent years, the outbreak of disease has resulted in severe mortality of scallops. The investigation of acetylcholinesterase in the cholinergic nervous system of *C. farreri* will help further cognizing the cholinergic nervous system in invertebrate, as well as its modulation to the innate immune system. The main objective of this study were (1) to characterize the structural feature of AChE gene from *C. farreri* (designated as CfAChE) and the activity of its recombinant protein, (2) to investigate the mRNA expression pattern of CfAChE in different tissues, (3) to detect its temporal expression in haemocytes against immune stimulation (LPS and TNF-α), and (4) to examine the effects of inhibitor DDVP on the activity of AChE in haemolymph, and the expression level of lysozyme mRNA in haemocytes to better understand the potential immunomodulation of CfAChE.

## Materials and Methods

### Ethics statement

The scallops used in the present study are marine cultured animals, and all the experiments are conducted according to the regulations of local and central government. The animal experiments were approved by the Animal Care and Use Committee at Qingdao institute for the control of drug products with a permit number of SCXX (Shandong) 20090007, which complied with the National Institute of Health Guide for the Care and Use of Laboratory Animals.

### Treatments and sample collection

All scallops *C. farreri* employed in the experiments were collected from a Haizhenpin farm in Qingdao, Shandong Province, China, and acclimatized in the aerated seawater for two weeks before processing. Seven tissues of six healthy scallops were sampled for total RNA extraction, including haemolymph, kidney, gill, hepatopancreas, adductor muscle, gonad and mantle.

One hundred and twenty scallops were employed for the LPS stimulation experiment in May 2010. Fifty scallops of them received an injection of 50 µL phosphate buffered saline (PBS, 377 mmol L^−1^ NaCl, 2.7 mmol L^−1^ KCl, 8.09 mmol L^−1^ Na_2_HPO_4_, 1.47 mmol L^−1^ KH_2_PO_4_, pH 7.4, osmolarity 780 mOsm L^−1^) were employed as the control group. Other 50 scallops received an injection of 50 µL LPS from *Escherichia coli* 0111:B4 (Sigma Aldrich, 0.5 mg mL^−1^ in PBS) were employed as the LPS stimulation group [37]. These 100 scallops were returned to water tanks after treatment, and 6 individuals were randomly sampled at 3, 6, 12, 24, 48 and 96 h post-injection. The rest twenty scallops were employed as the blank group, and 6 individuals were randomly sampled at 0 h. The haemolymph was collected, and then centrifuged at 800× g, 4°C for 10 min to harvest the haemocytes for RNA extraction.

Another one hundred and forty five scallops were employed for the TNF-α stimulation experiment in October 2010, and divided into four groups, control group, TNF-α stimulation group 1, TNF-α stimulation group 2, and blank group. There were 135 scallops in the first three groups, and forty-five scallops in each group received an injection of 50 µL of PBS, 5.0 ng mL^−1^ human TNF-α (Invitrogen, in PBS), 50.0 ng mL^−1^ TNF-α, respectively. These treated scallops were returned to water tanks, and six individuals were randomly sampled at 1, 3, 6, 9 and 12 h post-injection from control and stimulation groups. Simultaneously, ten scallops were randomly sampled at 0 h in the blank group containing untreated scallops. The haemolymph were collected and stored as described above.

Two hundred and fifty scallops employed for DDVP stimulation experiment were collected in December 2010. After two weeks acclimation, two hundred scallops were cultured in the aerated seawater containing 50 mg L^−1^ DDVP, and they were employed as the DDVP stimulation group. The rest fifty scallops without DDVP treatment were employed as the blank group. Six individuals were randomly sampled at 0, 3, 6, 12, 24, 48 and 96 h after DDVP treatment. Haemolymph from six scallops was also collected from the adductor muscle using a syringe, and then immediately centrifuged at 800×g, 4°C for 10 min to collect the supernatant for AChE activity assay and haemocytes for RNA extraction.

### Cloning the full-length cDNA of CfAChE

BLAST analysis of the scallop EST sequences revealed that one contig (no. rscag0 001935; length: 452 bp) was homology to the previously identified AChE [38]. Five gene specific primers, sense primer P1, P2, P3 and reverse primer P4, P5 (Table 1), were designed based on this EST to clone the full-length cDNA of CfAChE by rapid amplification of cDNA ends (RACE) approach. PCR amplification to clone the 3′ end of CfAChE was carried out using sense primer P1, P2 or P3 and antisense primer Oligo(dT)-adaptor P6, while sense primer Oligo(dG)-adaptor P7 and antisense primer P4 or P5 were used to get the 5′ end according to the Usage Information of 5′ RACE system (Invitrogen). All PCR amplification was performed in a PCR Thermal Cycle (TAKARA, GRADIENT PCR).

**Table 1:** Sequences of the primers used in the experiment.

Primer	Sequence (5′-3′)	Sequence information
P1 (forward)	TCACTTCAATCTTAGCGTCAG	CfAChE specific primer
P2 (forward)	CGACGGGAAACTTGAGATTGGCT	CfAChE specific primer
P3 (forward)	GGTAATGGTTTGGATACACGGAG	CfAChE specific primer
P4 (reverse)	CTGACGCTAAGATTGAAGTGA	CfAChE specific primer
P5 (reverse)	AGCCAATCTCAAGTTTCCCGTCG	CfAChE specific primer
P6 (forward)	GGCCACGCGTCGACTAGTACT17	Oligo(dT)-adaptor
P7 (reverse)	GGCCACGCGTCGACTAGTACG10	Oligo(dG)-adaptor
P8 (forward)	GGCATCATTAAGATTCTTAATATCAGATG	CfAChE ORF primer
P9 (reverse)	GGACCAACTTTGCAAAAACAGGG	CfAChE ORF primer
P10 (forward)	GAATTCTCAGTTACCTGCGATGGCAATGCGCG	Recombination primer
P11 (reverse)	GCGGCCGCCCCTGTTTTTGCAAAGTTGGTCC	Recombination primer
P12 (forward)	TATCGCAGAGTGGAAGCCCT	Real-time CfAChE primer
P13 (reverse)	GTTGTTGAGCAGCATAGCAGGAT	Real-time CfAChE primer
P14 (forward)	ATCCGCTACCACTCCTG	Real-time lysozyme primer
P15 (reverse)	CTGACCGATGTATGCCAC	Real-time lysozyme primer
P16 (forward)	CAAACAGCAGCCTCCTCGTCAT	Real-time actin primer
P17(reverse)	CTGGGCACCTGAACCTTTCGTT	Real-time actin primer
M13-47	CGCCAGGGTTTTCCCAGTCACGAC	Vector primer
RV-M	GAGCGGATAACAATTTCACACAGG	Vector primer
5′AOX	GACTGGTTCCAATTGACAAGC	Vector primer
3′AOX	GCAAATGGCATTCTGACATCC	Vector primer

The PCR products were gel-purified and cloned into pMD18-T simple vector (Takara, Japan). After being transformed into the competent cells of *E. coli* Top10F, the positive recombinants were identified through anti-Ampicillin selection and PCR screening with sense vector primer RV-M and antisense vector primer M13-47 (Table 1). The positive clones were sequenced on an ABI 3730 XL Automated Sequencer (Applied Biosystems). The sequencing results were verified and subjected to cluster analysis.

After obtaining the full-length sequence of CfAChE, PCR amplification with sense primer P8 and antisense primer P9 (Table 1, designed according to CfAChE ORF sequence) was performed to verify the ORF of CfAChE.

### Sequence analysis

The similarity searches were performed with the BLAST program at the National Center for Biotechnology Information (http://www.ncbi.nlm.nih.gov/blast). The deduced amino acid sequence was analyzed with the Expert Protein Analysis System (http://www.expasy.org). SignalP 3.0 program was utilized to predict the presence and location of signal peptide, and the cleavage sites in amino acid sequences (http://www.cbs.dtu.dk/services/SignalP). The protein domain was predicted by Simple Modular Architecture Research Tool (SMART) version 5.1 (http://smart.Embl-heidelberg.de/). Multiple sequences alignment of CfAChE with other AChEs was performed with the ClustalW multiple alignment program (http://www.ebi.ac.uk/clustalw/) and multiple alignment show program (http://www.biosoft.net/sms/index.html). The intramolecular disulfide was predicted by DiANNA 1.1 web server (http://clavius.bc.edu/~clotelab/DiANNA/). An unrooted phylogenic tree was constructed based on the deduced amino acid sequences of CfAChE and other known AChEs by the Neighbor-Joining (NJ) algorithm using the MEGA4.1 software (http://www.megasoftware.net). To derive the confidence value for the phylogeny analysis, bootstrap trials were replicated 1000 times.

### Recombinant expression of CfAChE and protein purification

The cDNA sequence coding the mature CfAChE peptide was amplified with specific primers P10 and P11. For the convenience of cloning, an *Eco*RΙ site was added to the 5′ end of P10, and a *Not*Ι site was added to the 5′ end of primer P11. The purified PCR product was first cloned into pMD18-T simple vector (Takara, Japan), and digested with the restriction enzymes *Eco*RΙ and *Not*Ι, and then cloned into *Eco*RΙ/*Not*Ι site of the *Pichia pastoris* expression vector pPICZαA (Invitrogen). The resulting plasmid was transformed into *E. coli* DH5α for nucleotide sequencing to ensure in-frame insertion.

The constructed recombinant plasmid was linearized by *Pme*I and transformed into competent cell of *P. pastoris* GS115 with a Micro-Pulser electroporator (Bio-Rad, Beijing, China) as recommended by manufacturer's instructions (Invitrogen). The cells were then spread on YPDS plates (1% yeast extract, 2% peptone, 2% glucose, 2% agar, 1 mol L^−1^ sorbitol and 100 µg mL^−1^ Zeocin) for selection of positive clones.

The positive clones were inoculated into BMGY medium (1% yeast extract, 2% peptone, 100 mmol L^−1^ potassium phosphate, pH 6.0, 1.34% yeast nitrogen base, 4×10^−5^% biotin and 1% glycerol) and grew at 30°C until the culture reached OD_600_ = 2–6. The cells were harvested by centrifuging at 1500× g for 5 min, and cell pellet was resuspended in BMMY medium (1% yeast extract, 2% peptone, 100 mmol L^−1^ potassium phosphate, pH 6.0, 1.34% yeast nitrogen base, 4×10^−5^% biotin and 1% methanol) to 1.0 absorbance at 600 nm. To induce the expression, methanol was added every 24 h to a final volume concentration of 1% for successive 4 days. The supernatant was obtained by centrifugation (6000 g for 1 min) and further purified by nickel affinity chromatogramphy MagExtractor His-Tag NPK-700 (Toyobo) under native condition as described by the manufacturer. The purified recombinant CfAChE (designed as rCfAChE) was detected by 12% SDS-polyacrylamide gel (SDS-PAGE).

### Deglycosylation of the CfAChE by PNGase F

PNGase F (NEB) was used to deglycosylate the purified rCfAChE. Ten microgram of rCfAChE was added into 2 µL Glycoprotein Denaturing Buffer (5% SDS, 400 mmol L^−1^ DTT), and then the mixture was incubated at 100°C for 10 minutes. After the addition of 2 µL NP-40, 2 µL G7 Reaction Buffer (50 mmol L^−1^ sodium phosphate, pH 7.5), 1 µL PNGase F and 3 µL H_2_O, the reaction mixture was incubated at 37°C for 1 hour to deglycosylate rCfAChE. Finally, the reaction product was visualized by SDS-PAGE.

### Bioassay of rCfAChE activity

The concentration of rCfAChE was determined according to Bradford's description [39], and the CfAChE activity was determined in triplicate for each sample according to the colorimetric method initially ameliorated by Xuereb et al [40]. Firstly, 330 µL of phosphate buffer (0.1 mol L^−1^, pH 7.8), 20 µL of the chromogenic agent 5,5′-Dithio bis-(2-nitrobenzoic acid) (DTNB) (0.0076 mol L^−1^) and 20 µL of rCfAChE (1.52 µg mL^−1^) were added to a 96-well microtiter plate. For the sample in the DDVP stimulation experiment, rCfAChE was substituted for 50 µL of the supernate of haemolymph. Measurement of enzyme activity was initiated after the addition of 10 µL acetylthiocholine iodide (ATC) solution (0.076 mol L^−1^). Spontaneous substrate hydrolysis was assessed in two controls without ATC and sample protein, respectively. Absorption of resultant 2-nitro-5-thiobenzoate anion was recorded at 405 nm every 5 min for 15 min (25°C) using a precision microplate reader (Emax). The velocity data (ΔOD min^−1^) were converted to specific AChE activity (µmol min^−1^ mg^−1^ protein) as follows: (ΔOD min^−1^×3.8×10^−4^ L)/(ε×[E_t_]), where ε = 1.36×10^4^ L mol^−1^ cm^−1^ and [E_t_] = 1.52 µg mL^−1^. One unit of AChE activity was defined as the amount of enzyme hydrolyzing 1 µmol of ATC in 10 min.

### RNA isolation, cDNA synthesis and SYBR Green fluorescent quantitative real-time PCR

Total RNA was extracted from different tissues of six healthy scallops, and the haemocytes from the scallops in the LPS, TNF-α and DDVP stimulation experiment, according to the protocol of Trizol (Invitrogen). MLV reverse transcriptase (Promega) was used to synthesize single-strand cDNA with Oligo(dT)-adaptor primer (Table 1) after the DNaseI (Promega) treated total RNA. The mixture was incubated at 42°C for 1 h, then heated at 95°C for 5 min to terminate the reaction, and subsequently stored at −80°C for the SYBR Green fluorescent quantitative real-time PCR (RT-PCR).

The quantitative RT-PCR amplification was carried out in an ABI 7300 Real-time Thermal Cycler according to the manual (Applied Biosystems) [41]. Two CfAChE gene-specific primers (P12 and P13, Table 1) and two lysozyme gene-specific primers (P14 and P15, Table 1) were used to amplify products of 269 bp and 93 bp from *C. farreri* cDNA template respectively, and the PCR products were sequenced to verify the specificity of RT-PCR. Two β-actin primers, P16 and P17 (Table 1), were used to amplify a 94 bp fragment as an internal control to verify the successful transcription and to calibrate the cDNA template for corresponding scallop samples. Dissociation curve analysis of amplification products was performed at the end of each PCR to confirm that only one PCR product was amplified and detected. After the PCR program, data was analyzed using ABI 7300 SDS software V2.0 (Applied Biosystems). To maintain consistency, the baseline was set automatically by the software. The 2^−ΔΔ*C*T^ method was used to analyze the expression level of CfAChE [42].

### Statistical analysis

All data was given as means ± S.E. The data was subjected to one-way analysis of variance (one-way ANOVA) followed by a multiple comparison (S-N-K). Difference was considered significant at *P*<0.05.

## Results

### The molecular characters of CfAChE cDNA

The complete sequence of CfAChE cDNA was obtained by overlapping EST racag0_001935 with the amplified fragments, and deposited in GenBank under accession no. **JN418764**. It consisted of a 5′ terminal untranslated region (UTR) of 108 bp, a 3′ UTR of 89 bp with a poly (A) tail, and an ORF of 1569 bp. The ORF encoded a polypeptide of 522 amino acids with a theoretical isoelectric point of 6.26 and predicted molecular weight of 58.3 kDa.

**Figure 1:**
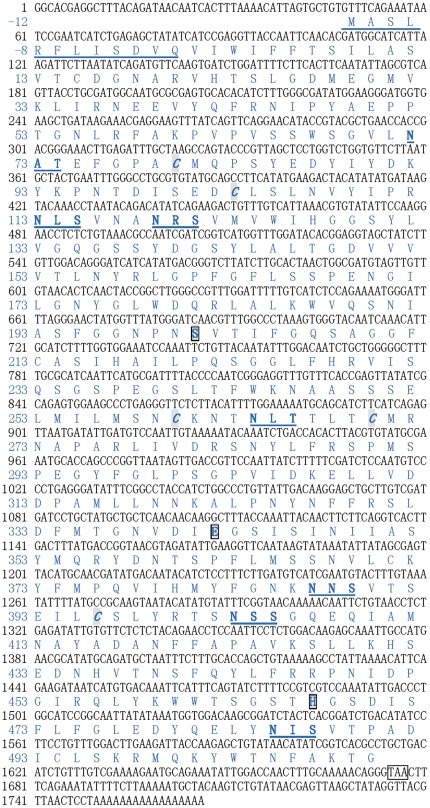
Nucleotide and deduced amino acid sequences of CfAChE. The amino acid residues in the mature protein were assigned positive numbers, and those in signal peptide were assigned negative numbers. The stop codon was labeled with box. The nucleotides and amino acids were numbered along the left margin. The predicted *N*-glycosylation sites were bold and underlined. The conserved cysteine residues were marked in italic and grey. The three residues forming the catalytic triad, S220, E354, and H479, were grey and labeled with box.

There were seven putative *N*-glycosylation sites in the deduced amino acid sequence of CfAChE (Fig. 1). A signal peptide of twelve residues was predicted at the N-terminus of CfAChE by SignalIP software. A coesterase domain was identified in CfAChE from Val13 to Gly522, and two pairs of disulfide (Cys92-Cys115 and Cys272-Cys282) were predicted by SMART program analysis.

### Multiple sequences alignment and phylogenic analysis

**Figure 2:**
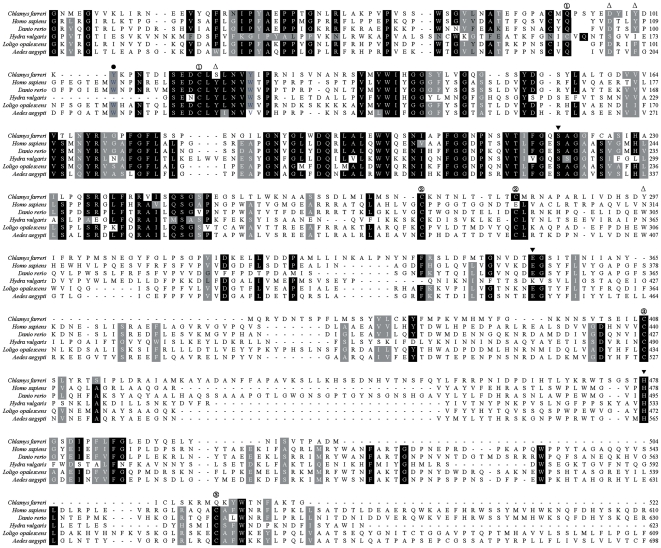
Multiple sequences alignment of CfAChE with other AChEs deposited in GenBank. The CfAChE amino acid sequence was aligned with AChEs from *Hydra vulgaris* (CAA06981), *Loligo opalescens* (AAD15886), *Aedes aegypti* (NP_476953), *Danio rerio* (AAC14022) and *Homo sapiens* (NP_000656). The amino acid numbers for each sequence were indicated at right. The AChE catalytic triad residues were indicated by filled triangle. The PAS site was labeled by hollow triangle. The choline binding site was labeled by filled circles. And the intramolecular disulfide bonds were indicated by number and open circles.

A search for homology of the deduced amino acid sequence of CfAChE with other AChEs in GenBank/EMBL Data Bank revealed that CfAChE shared low identity with other AChEs, such as 21.4% identity with *Hydra vulgaris* (AAC14022), 22.1% with *Loligo opalescens* (AAD15886), 29.0% with *Rhipicephalus appendiculatus* (CAA06981), 25.6% with *Torpedo Californica* (NP_476953) and 23.7% with *Homo sapiens* (NP_000656). The conserved structure features of AChEs were identified in CfAChE, including the catalytic site triad consisting of three residues (Ser220, Glu354, and His479), the choline binding site (Tyr105), the PAS site (Asp99, Tyr102, Ser117, and Tyr297), and 5 cysteine residues forming two intramolecular disulfide bonds (Cys92 and Cys115, Cys272 and Cys282) (Fig. 2).

**Figure 3:**
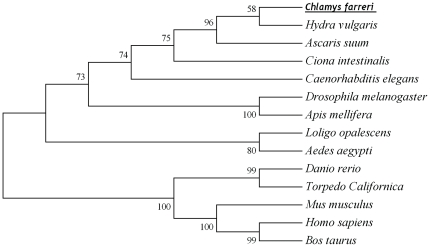
Consensus neighbor-joining tree based on the sequences of CfAChE and other AChEs from different animals. The protein sequences used for phylogenetic analysis include: *Hydra vulgaris* (CAA06981), *Ascaris suum* (ADY44392), *Ciona intestinalis* (NP_001122349), *Caenorhabditis elegans* (AAC14022), *Drosophila melanogaster* (NP_476953), *Apis mellifera* (NP 001035320), *Loligo opalescens* (AAD15886), *Aedes aegypti* (NP_476953), *Danio rerio* (AAC14022), *Torpedo Californica* (P04058), *Mus musculus* (NP 033729), *Bos taurus* (NP 001069688) and *Homo sapiens* (NP_000656).

Based on the amino acids alignment, a phylogenic tree was constructed by using MEGA program (Fig. 3). Vertebrate and invertebrate AChEs were positioned separately as two distinct groups in the phylogenic tree. CfAChE was first clustered with AChEs from cnidaria and nematoda (*Hydra vulgaris*, *Ascaris suum* and *Caenorhabditis elegans*), and then gathered together with AChEs from mollusc and arthropod to form the first group (*Loligo opalescens*, *Aedes aegypti*, *Apis mellifera* and *Drosophila melanogaster*). All the AChEs from chordates, including *Torpedo Californica*, *Danio rerio*, *Mus musculus*, *Bos taurus*, *Homo sapiens*, were clustered together and formed a sister group to the first group.

### The activity of rCfAChE

**Figure 4:**
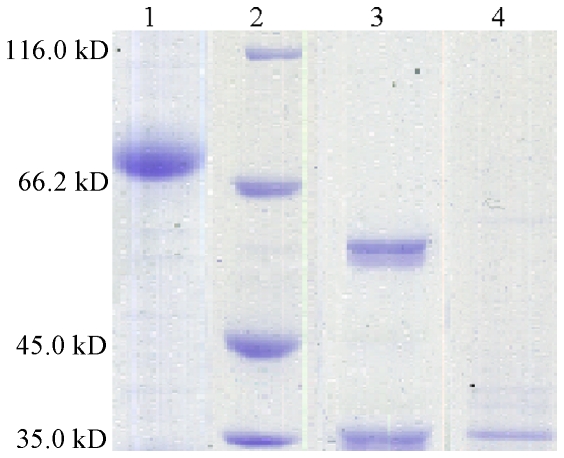
SDS-PAGE analysis of recombinant CfAChE. After electrophoresis, the gel was visualized by Coomassie brilliant blue R250 staining. Lane 1: purified recombinant CfAChE; Lane 2: protein molecular standard; Lane 3: recombinant CfAChE after deglycosylation by PNGase F; Lane 4: PNGase F as control.

The purified rCfAChE migrated as a broad band at around 70 kDa on SDS-PAGE. After deglycosylation, the molecular weight of rCfAChE was around 58 kDa, which was close to the predicted theoretical molecular weight (Fig. 4). The activity of purified rCfAChE was examined through AChE activity assay. The specific activity of rCfAChE was 71.3±1.3 U mg^−1^ against the substrate ATC.

**Figure 5:**
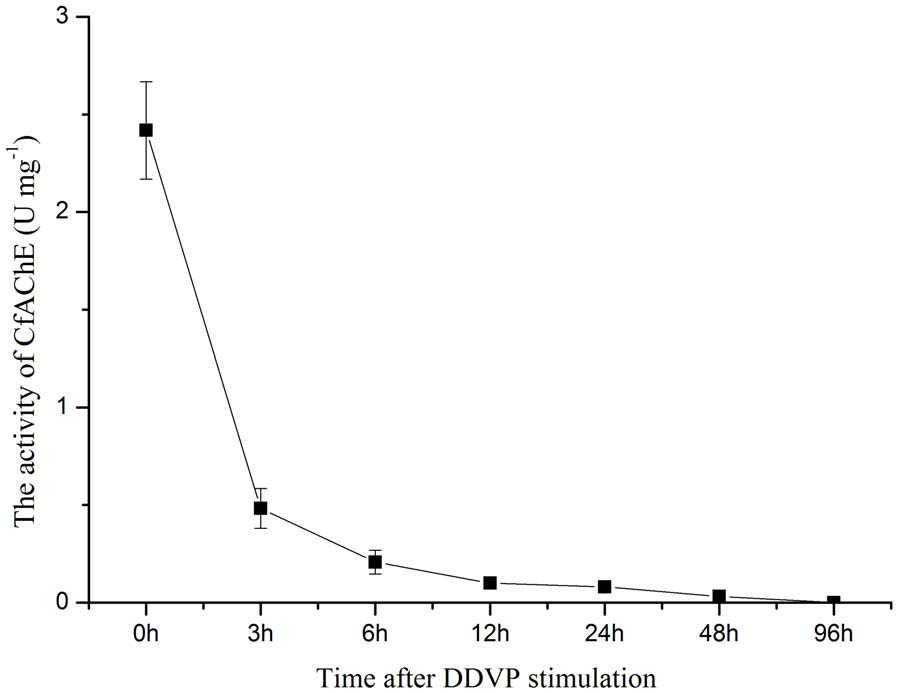
The activity of CfAChE in serum after DDVP stimulation for 0, 3, 6, 12, 24, 48 and 96 h. The CfAChE activities were determined in each group and the unstimulated scallops were used as the blank groups. Values were shown as mean ± S.E., N = 6.

In DDVP stimulation experiment, the activity of rCfAChE was determined in the supernatant sample during the period of 96 h after stimulation (Fig. 5). The activity of CfAChE was determined to be 0.16 U mg^−1^ in the blank group at 0 h, but it dropped to 0.03 U mg^−1^ (P<0.05) at 3 h after DDVP stimulation. Then it decreased gradually to undetectable level from 6 to 96 h after DDVP stimulation.

### Tissue distribution of CfAChE mRNA

**Figure 6:**
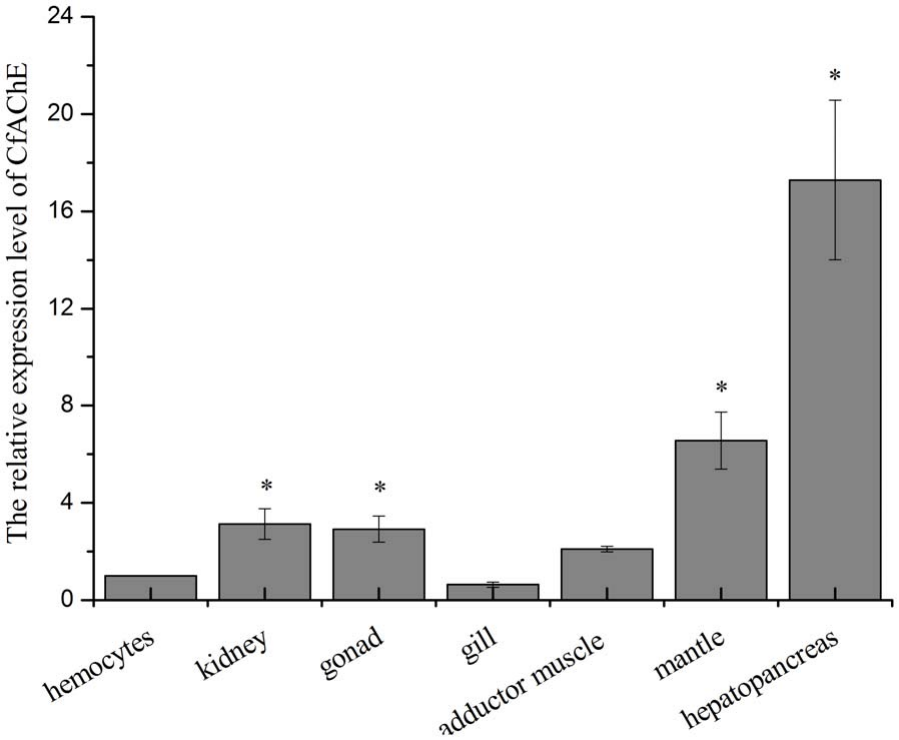
Tissue distribution of the CfAChE transcripts detected by SYBR Green RT-PCR. CfAChE transcript level in adductor muscle, mantle, gill, hepatopancreas, kidney and gonad of six adult scallops was normalized to that of haemocytes. Vertical bars represented the mean ± S.E. (N = 6), and bars with * are significantly different (*P*<0.05).

The expression level of CfAChE mRNA was examined in the healthy scallops with β-actin as internal control. For CfAChE and β-actin genes, there was only one peak at the corresponding melting temperature in the dissociation curve analysis, indicating that the PCR was specifically amplified (data not shown). The mRNA transcripts could be detected in all the tested tissues, including haemocytes, hepatopancreas, kidney, adductor muscle, gonad, gill and mantle. The highest CfAChE expression level was found in hepatopancreas, which was 12.9-fold of that in haemocytes (P<0.05), and it was significantly higher than that in other tissues (P<0.05). The expression of CfAChE in mantle, kidney and gonad was 6.6, 3.1 and 2.9-fold of that in haemocytes, respectively (P<0.05). The CfAChE mRNA transcripts in gill were in the lowest level. There was no significant difference in the mRNA transcripts between haemocytes and adductor muscle (P>0.05) (Fig. 6).

### The temporal expression of CfAChE mRNA in haemocytes after LPS and TNF-α stimulation

**Figure 7:**
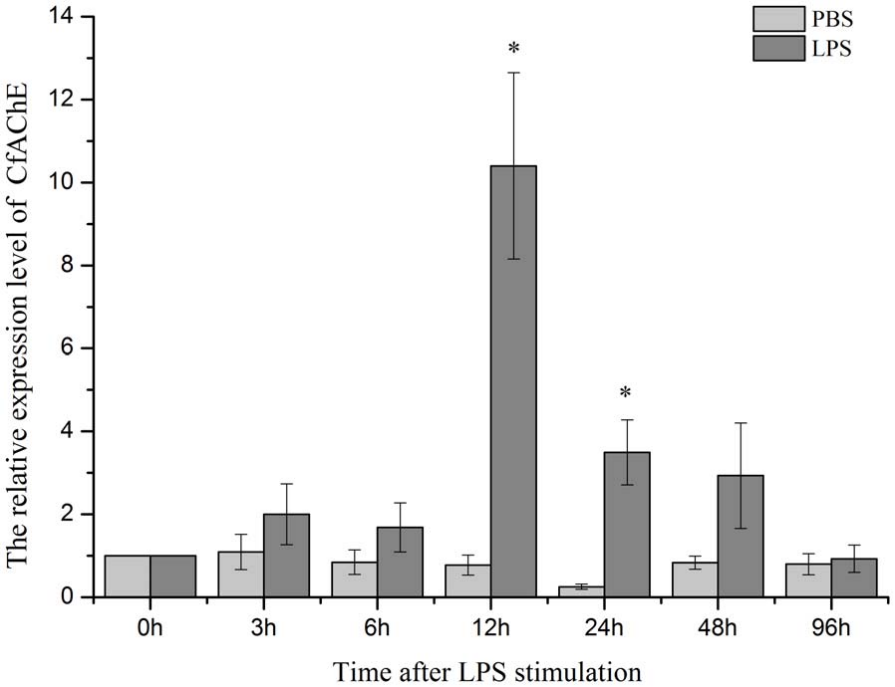
The temporal expression of CfAChE mRNA in haemocytes after LPS stimulation for 3, 6, 12, 24, 48 and 96 h. Data was expressed as the ratio of the CfAChE mRNA to the β-actin mRNA. The relative CfAChE expression level was determined for each group, and the unstimulated scallops (0 h) were used as the blank group, and scallops injected with PBS were used as the control group. Values were shown as mean ± S.E. (N = 6), and bars with * are significantly different (*P*<0.05).

The expression level of CfAChE mRNA in haemocytes after LPS and TNF-α stimulation was quantified by quantitative real-time PCR. The level of CfAChE was up-regulated after LPS stimulation and reached the peak at 12 h, which was 10.4-fold of that in the blank group (P<0.05) (Fig. 7). Then the expression level of CfAChE mRNA decreased and it was 3.49-fold of that in the blank group at 24 h (P<0.05). There was no significant difference in the expression level of CfAChE mRNA between the stimulation and control group from 48 to 96 h after LPS stimulation (P>0.05).

**Figure 8:**
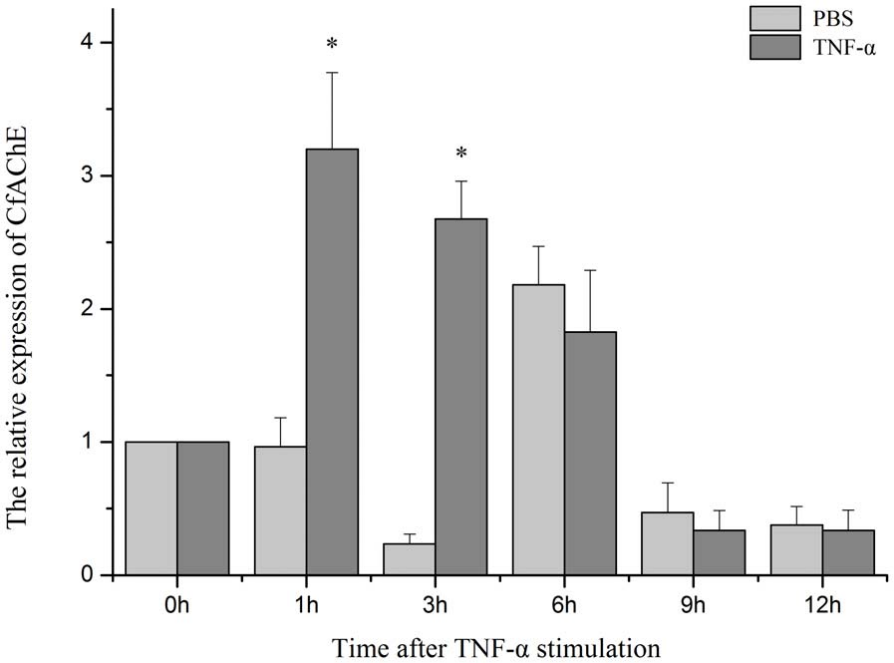
The temporal expression of CfAChE mRNA in haemocytes after TNF-α stimulation for 1, 3, 6, 9 and 12 h. Data was expressed as the ratio of the CfAChE mRNA to the β-actin mRNA. The relative CfAChE expression level was determined for each group, and the unstimulated scallops (0 h) were used as the blank group, and scallops injected with PBS were used as the control group. Values were shown as mean ± S.E. (N = 6), and bars with * are significantly different (*P*<0.05).

When scallops were stimulated with TNF-α, the expression level of CfAChE mRNA in haemocytes could not change after the stimulation of 5.0 ng mL^−1^ TNF-α (data was not shown), while it could be up-regulated obviously after 50.0 ng mL^−1^ TNF-α stimulation (Fig. 8). The expression level of CfAChE mRNA in the stimulation group increased significantly at 1 h and 3 h, which were 3.2 and 2.7-fold (P<0.05) of that in the blank group, respectively. No significant difference in the expression level of CfAChE mRNA was observed between the stimulation group and control group from 6 to 12 h after TNF-α stimulation (P>0.05).

### The temporal expression of lysozyme mRNA in haemocytes after DDVP stimulation

**Figure 9:**
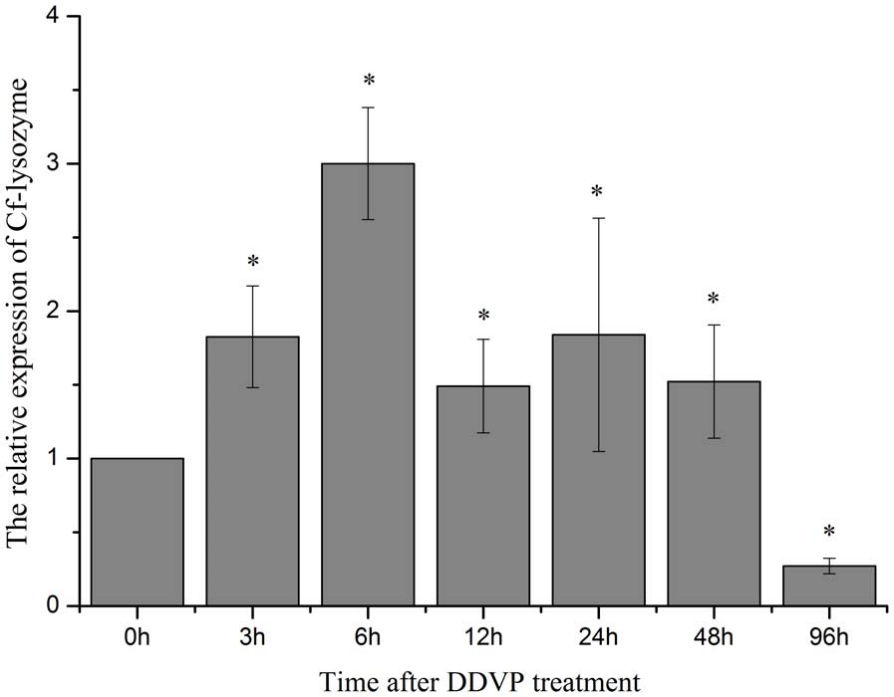
The temporal expression of lysozyme mRNA in haemocytes of *C. farreri* after DDVP treatment for 0, 3, 6, 12, 24, 48 and 96 h. Data was expressed as the ratio of the CfAChE mRNA to the β-actin mRNA. The relative CfAChE expression level was determined for each group and the unstimulated scallops were used as the control group. Values were shown as mean ± S.E. (N = 6), and bars with * are significantly different (*P*<0.05).

The expression level of lysozyme mRNA in haemocytes of scallops was up-regulated after DDVP stimulation (Fig. 9). The CfAChE expression level began to increase at 3 h (P>0.05), and reached the peak at 6 h, which was 3.0-fold of that in the blank group (P<0.05). Then it began to decrease and reached 1.5, 1.8 and 1.5-fold of that in the blank group at 12, 24 and 48 h (P<0.05), respectively. Finally, it decreased significantly to be 0.27-fold of that in the blank group at 96 h after DDVP stimulation (P<0.05).

## Discussion

The immune system could protect host against the invasive pathogenic organisms, which is known as innate and adaptive immunity in vertebrates, whereas only innate immunity in invertebrates. Acetylcholinesterase (AChE; EC 3.1.1.7) in vertebrates was involved in cell development and maturation [43], neuronal development and nerve regeneration [44] and inflammation modulation [45]. AChE had also been identified in most invertebrates, including mollusc [15], arthropoda [46], platyhelminthes [47], annelida [48] and nematoda [34]. And AChE was also reported to be involved in many behaviors in these invertebrates, including locomotion [34], [40], [49], feeding [34], [40], egg laying [34], male mating [34], embryo development [50] and digestive activity [15]. However, the immunomodulation of AChE is still unclear in invertebrates. In the present study, an AChE gene was identified from bivalve scallop *C. farreri*. The deduced protein of CfAChE was composed of 522 amino acids, and it shared 21.4–29.0% identity with other AChEs. CfAChE owned all the conserved key sites responsible for catalytic function, including the catalytic triad (Ser220, Glu354 and His479), the choline binding site (Tyr105) and the PAS site (Asp99, Tyr102, Ser117, and Tyr297) [51]–[53]. There were five conserved cysteines in CfAChE to form two disulfide bonds (Cys92–Cys115 and Cys272–Cys282), which was similar to AChEs from *Boophilus decoloratus*, *Rhipicephalus appendicalatus* [54] and *Locusta migratoria manilensis* [55], while different with most of AChEs with three conserved disulfide bonds constituted by six cysteines. In NJ phylogenic tree, CfAChE was firstly clustered with AChEs from invertebrates such as nematoda and mollusc, and then gathered together with AChEs from chordates. Considering the low sequence identity and the difference in the number of disulfide bonds, it was necessary to validate the CfAChE catalytic activity. In the activity assay, rCfAChE could hydrolyze ATC, and the activity was detected to be 71.3±1.3 U mg^−1^. The structural and functional results indicated strongly that CfAChE was the homologue of acetylcholinesterase in scallop *C. farreri*, and capable of catalyzing the hydrolysis reaction of ACh.

AChE plays a crucial role in the physiological adjustment activity of the cholinergic nervous system in vertebrate. The information on the tissue distribution of CfAChE might offer useful clues to investigate the function of CfAChE as well as the effect of ACh in scallops. In this study, CfAChE mRNA transcripts could be detected in all the examined tissues, including haemocytes, gill, gonad, muscle, kidney, mantle and hepatopancreas, with the highest level in hepatopancreas. The universal expression of CfAChE mRNA indicated that the hydrolysis of ACh by CfAChE might be essential to most physiological function of scallops. The highest expression level in the hepatopancreas suggested CfAChE was probably involved in immune or metabolic processes of scallops, because hepatopancreas played important role in innate immunity and metabolism of scallops [56]. The detection of CfAChE mRNA in gonad and gill indicated that it might participate in egg laying, male mating [34], and the ciliary movement in the gill plates [57]. The circulating haemocytes had been reported to participate in several physiological processes in bivalves, such as wound repair, shell repair, nutrient digestion and transport, excretion and immune defense [58], [59]. The mRNA transcript of CfAChE detected in haemocytes implied that CfAChE might be involved in the immune response of scallops.

The haemocytes of invertebrate animals play important roles in the innate immunity [60], [61]. In order to gain the further information about the functions of CfAChE in the immune response of scallops, its temporal expression in haemocytes, the main immunocytes [62]–[64], was determined after LPS stimulation. The expression level of CfAChE mRNA in haemocytes increased significantly after LPS stimulation at 12 h and 24 h, which were 10.4 and 3.49-fold (P<0.05) of that in the blank group, respectively, and then recovered to the normal level at 48 h and 96 h. While the mRNA expression in the control group did not change significantly during the whole experiment (Fig. 7). The results demonstrated that LPS stimulation induced the expression level of CfAChE in haemocytes, which was consistent with the observation that LPS stimulation resulted in the significant increase of AChE activity in the brain regions in vertebrate [65]. In vertebrate, LPS stimulation as immune challenge could induce the higher expression of the proinflammatory cytokines such as TNF-α and IL-1 to activate the cholinergic nervous system, which could release acetylcholine to down-regulate the immune response through cholinergic ion channel and cholinergic anti-inflammatory pathway [5], [29], [66], and then high concentration of acetylcholine in blood could induce the expression of AChE in immunocytes to return the homeostasis [5], [29]. ACh and its receptors as well as some cytokines had been identified and characterized in mollusk. For example, a TNF-α homologue had been characterized in disk abalone *Haliotis discus discus* [67], and ACh and nicotinic ACh receptor subunits had been identified in the haemolymph of *Aplysia californica* and the nervous system of *Lymnaea stagnalis*, respectively [68], [69]. Considering the presence of these components in mollusc, it was suspected that a similar activation mechanism as vertebrates probably existed in scallops to induce the expression of CfAChE in haemocytes after LPS stimulation.

In order to further confirm the activation mechanism and immunomodulation of CfAChE in the haemolymph, the TNF-α stimulation was introduced to detect the temporal expression of CfAChE mRNA in the haemocytes of scallop. TNF-α was an important mediator in innate immunity and could be activated by LPS stimulation [70]. In the present study, the expression level of CfAChE mRNA could not be induced by low dose of TNF-α (5.0 ng mL^−1^), but increased significantly at 1 h and 3 h after the high dose of TNF-α (50 ng mL^−1^) stimulation, which was 3.2 and 2.7-fold (P<0.05) of that in the blank group respectively, and then it recovered to the normal level from 6 h to 12 h (P>0.05) (Fig. 8). The result suggested that high dose of cytokine TNF-α could induce the expression of CfAChE mRNA in scallops, and further explain the higher expression of CfAChE stimulated by LPS. The high expressed CfAChE would decrease the concentration of ACh in haemolymph, and retard the negative modulation of ACh on the immune response [33]. The negative immunomodulation of ACh was very important to decrease the immunologic injury and maintain the immune homeostasis. However, the repression of the negative immunomodulation was also required at a suitable or opportune time, because lower immune level would increase the susceptibility to other pathogens. These results indicated that CfAChE mRNA expression could be induced by cytokines during the immune response, and it might hydrolyze ACh to repress the negative immunomodulation of ACh and rebuild the homeostasis destroyed by immune response.

The inhibition of AChE activity could increase ACh for the ligation with cholinergic receptors on immune cells and suppress several inflammatory reactions such as cytokines production, T-cell proliferation and CNS inflammatory in vertebrate [33]. In this study, the irreversible AChE inhibitor DDVP was selected to further study the role of CfAChE in the immunomodulation. After DDVP treatment, the CfAChE activity in the supernatant of haemolymph was inhibited significantly, which dropped from 0.16 to 0.03 U mg^−1^ at the first 3 h, and reached finally to the undetectable level at 96 h (Fig. 5). The result was consistent with the observation that AChE inhibitor could reduce the extracellular AChE activity effectively in vertebrate [33]. Decreased AChE activity would slow down the hydrolysis reaction of ACh and increase the concentration of ACh in haemolymph of scallops, which would become one vital modulator of the downstream immune response. The accumulation of ACh might inhibit the production of cytokines, and the low level of cytokines increased the expression of immune effectors. It was obvious that the expression of lysozyme mRNA was up-regulated from 3 to 48 h with the highest level at 6 h after the DDVP treatment (P<0.05) (Fig. 9). The result was consistent with the reports that cytokines could reduce the lysozyme activity in GG2EE macrophage cell line [71] and ACh could increase the activity of lysozyme in the muscle homogenates of rat [72]. Therefore, these results collectively indicated the inhibition of CfAChE would regulate immune response of scallop positively in the non-challenged situation, and the normal modulation of CfAChE activities was very important to the immune homeostasis in scallops.

In conclusion, an AChE gene was identified from scallop *C. farreri*, and its mRNA transcripts were ubiquitously distributed in haemocytes, adductor muscle, gill, hepatopancreas, mantle and gonad. The treatments with LPS, TNF-α and DDVP all up-regulated the expression of CfAChE. The results suggested that CfAChE probably played the similar roles of AChE in neuroimmune system of vertebrates, and it might help the scallop to maintain the homeostasis after immune response through catalyzing the hydrolysis of ACh.
